# Effect of finger rest positions on upper limb muscle activity during pre-clinical procedures

**DOI:** 10.7717/peerj.15663

**Published:** 2023-07-26

**Authors:** Júlia Margato Pazos, Mariana Segnini Tiberti, Simone Cecilio Hallak Regalo, Lívia Nordi Dovigo, Patricia Petromilli Nordi Sasso Garcia

**Affiliations:** 1 Department of Social Dentistry, School of Dentistry of Araraquara, Universidade Estadual Paulista, Araraquara, São Paulo, Brazil; 2 Department of Basic and Oral Biology, School of Dentistry of Ribeirão Preto, Universidade de São Paulo, Ribeirão Preto, São Paulo, Brazil

**Keywords:** Finger rest position, Electromyography, Occupational health, Ergonomics, Dentistry, Dental students

## Abstract

**Objectives:**

This study aimed to observe the effect of different finger rest positions on the muscular activity of the hand, forearm, arm, shoulder, thorax, and neck, as well as on the angular deviation from the neutral position of the neck, trunk, upper arm, and forearm on the working side during pre-clinical procedures.

**Methods:**

An experimental laboratory study was performed. Response variables were muscle activation of the abductor pollicis, brachioradialis, biceps brachii, deltoid, pectoralis major, and right sternocleidomastoid muscles and angular deviation from the neutral position of the neck, trunk, arm, and forearm during simulated clinical procedures. Independent variable was finger-rest position during cavity preparation (no finger rest, usual rest, and ergonomic rest). Class I cavity preparations (N = 120) were performed on artificial first molars (16, 26, 36, and 46) (N = 120). Muscular activation was assessed by surface electromyography and angular deviations using Software for Postural Assessment (SAPO) version 0.69. One-way analysis of variance and Tukey’s or Games-Howell’s *post-hoc* tests were performed (α = 0.05).

**Results:**

For the sternocleidomastoid muscle, there was no statistically significant difference between the different rest positions. For the deltoid muscle, work with no finger rest resulted in greater muscle activation (*p* < 0.001) during work on tooth 36. Regarding the pectoralis major and right brachioradialis muscles, we observed that for both teeth 16 and 26, working with ergonomic rest showed less muscle activation. Muscle activation of the right biceps brachii was higher for work with no rest in both the upper and lower arches, differing significantly only from the usual rest in tooth 16 (*p* < 0.001), usual rest and ergonomic rest in teeth 26 and 46 (*p* < 0.001), and only ergonomic rest in tooth 36 (*p* = 0.044). In the right abductor pollicis muscle, work with ergonomic rest resulted in less muscle activation for cavity preparation in teeth 16, 26, and 36, which was significantly different from work with no rest (*p* = 0.029, *p* < 0.001, and *p* = 0.013, respectively). Regarding angular deviation, it was observed that for tooth 16, there was a greater angular deviation of the arm when performing cavity preparations with no finger rest. For teeth 26 and 46, the ergonomic finger rest provided lower angular deviation from the neutral position of the right arm. For tooth 36, ergonomic rest provided less angular deviation from the neutral neck position.

**Conclusion:**

In general, the use of non-active finger rest during simulated cavity preparations, regardless of the type of rest, provided less muscle activation and angular deviation from the neutral position of the body’s upper extremity when performing pre-clinical procedures.

## Introduction

Musculoskeletal disorders are the most common and debilitating disorders associated with dental practice (Dable et al., 2014; Plessas & Bernardes Delgado, 2018; Hayes et al., 2021; Pazos et al., 2022). They consist of injuries that occur in muscles, tendons, nerves, vessels, joints, and the entire musculoskeletal system (Graça, Araújo & Silva, 2006), thereby causing severe pain and loss of efficiency and accuracy in clinical practice (Hoerler et al., 2012). In extreme cases, due to the resulting level of disability, these disorders can cause abandonment of professional careers (Garcia et al., 2012). Risk factors for musculoskeletal disorders include static postures maintained for a long period of time, repetitive movements performed routinely, incorrect posture, and difficulty in visualizing the work field, among others (Onety et al., 2014), and they may be present during pre-clinical training (Dable et al., 2014; Plessas & Bernardes Delgado, 2018; Pazos et al., 2022).

During this learning stage, holding instruments incorrectly and adopting awkward postures, such as trunk and neck twisting, exaggerated neck bending, and improper distance from the operative field often occur (Cosaboom-FitzSimons et al., 2008; Pazos et al., 2022; Garcia, Wajngarten & Campos, 2018; Garcia et al., 2012; Corrocher et al., 2014), as well as performing repetitive and vigorous movements with the hands, high pinching force, uncomfortable position of the wrists, and handling vibrating devices (Rempel et al., 2012; Dable et al., 2014; Arnett et al., 2017). To ensure greater precision and stability of the working hand during the development of their manual dexterity, students often work with the musculature of the upper limbs contracted and deviated from the neutral position (Dong et al., 2005). This behavior, if maintained over time, promotes muscle overload in this region, thereby leading to musculoskeletal disorders.

To minimize these factors, ergonomics is recommended to be included in dentistry courses (Corrocher et al., 2014; Alhazzazi et al., 2016; Cervera-Espert, Pascual-Moscardó & Camps-Alemany, 2018; Samoladas et al., 2018; Garcia, Wajngarten & Campos, 2018). Ergonomics is a science concerned with the health and welfare of workers, thereby promoting an adequate and comfortable workspace (Diaz-Caballero, Gómez-Palencia & Díaz-Cárdenas, 2010; Garcia et al., 2021). Teaching dental students about ergonomic working postures during the development of their manual dexterity is important for creating a healthy postural habit in this initial work phase to maintain occupational health throughout their professional lives (Dable et al., 2014; Arnett et al., 2017; Garcia, Wajngarten & Campos, 2018).

Furthermore, strategies, such as the use of non-active fingers as a resting position on a hard tissue (tooth or bone) in the oral cavity during the execution of procedures help to stabilize the working hand (Porto, 1994), promote greater precision and prevent sudden movements, and reduce muscle stress on the operator’s upper limbs (Dong et al., 2005; Cosaboom-FitzSimons et al., 2008). Although the use of finger rest has been recommended for many years (Dong et al., 2005; Cosaboom-FitzSimons et al., 2008), few scientific studies have proven which finger rest positions can provide greater protection against the development of musculoskeletal disorders in a dentist’s upper limbs (Dong et al., 2005).

The only studies related to this topic have evaluated the effect of finger rest on muscle activity during periodontal scaling (Dong et al., 2005; Cosaboom-FitzSimons et al., 2008). To the best of our knowledge, our study is the first to assess the impact of this technique on muscle activity during restorative procedures (cavity preparation), which are widely performed in the daily routine of dentists. Research that addresses this issue can make a great contribution to the field of occupational health, thereby helping to provide answers to the existing gap in this area.

Therefore, this study aimed to observe the effects of different finger rest positions on muscle activity and angular deviation from the neutral position of the upper extremity of the body during pre-clinical cavity preparations.

## Materials and Methods

### Study design

This study was approved by the Research Ethics Committee of São Paulo State University (UNESP), School of Dentistry, Araraquara, Brazil (CAAE Registry No. 50703021.7.0000.5416), and written informed consent was obtained from all participants.

This was an experimental laboratory study. Response/dependent variables included: (1) muscular activity of the hand, forearm, arm, shoulder, thorax, and neck on the working side, measured using surface electromyography (EMG); and (2) angular deviation from the neutral position of the neck, trunk, arm, and forearm, measured by the “Software for Postural Assessment,” while performing simulated clinical procedures (cavitary preparations). Independent variable was three different types of finger rest positions used during cavity preparation.

The sampling unit in this study was the pre-clinical cavity preparation, and the minimum sample size was determined using data from a pilot study with 80% power and 5% significance level. This resulted in 10 teeth for each experimental condition. The teeth and finger rest positions were randomized so that 10 cavity preparations of each tooth were performed (16, 26, 36, and 46) with each type of finger rest position (N = 40; N = 120). The responses and independent variables of each tooth were considered for further analysis.

### Simulated pre-clinical procedure (cavity preparation)

To simulate the clinical environment, a dental phantom head (MOM brand; Manequins Odontológicos Marília) was attached to the dental chair, which has artificial resin teeth that are specific for pre-clinical cavity preparation. Using a #1014 diamond burr at low speed, class I cavity preparations were made for composite resin in the right upper first molar (16), left upper first molar (26), left lower first molar (36), and right lower first molar (46). As the artificial tooth was prepared, it was replaced with a new intact tooth for the next cavity preparation.

### Finger rest positions

Three different forms of finger rest positions were proposed during the cavity preparations: (1) no finger rest; (2) usual finger rest; and (3) ergonomic finger rest, which followed the recommendations of the Discipline of Ergonomics in Dentistry, São Paulo State University (UNESP), School of Dentistry, Araraquara. A micromotor and contra-angle were held using a tripod grasp (thumb, index, and middle fingers) with the working hand.

**Figure 1:**
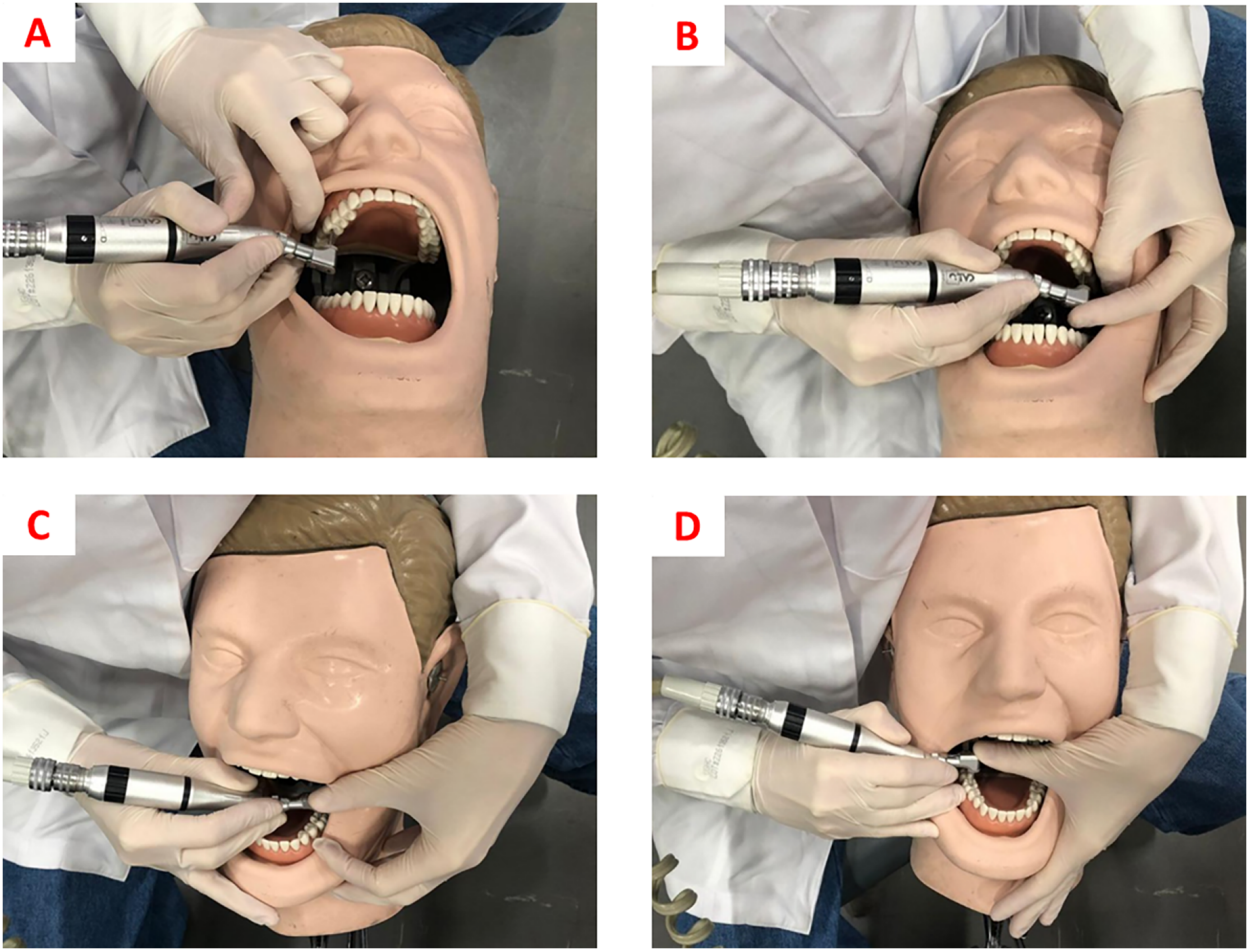
(A) Finger rests for cavity preparation on tooth 16. (B) Finger rests for cavity preparation on tooth 26. (C) Finger rests for cavity preparation on tooth 36. (D) Finger rests for cavity preparation on tooth 46.

**Figure 2:**
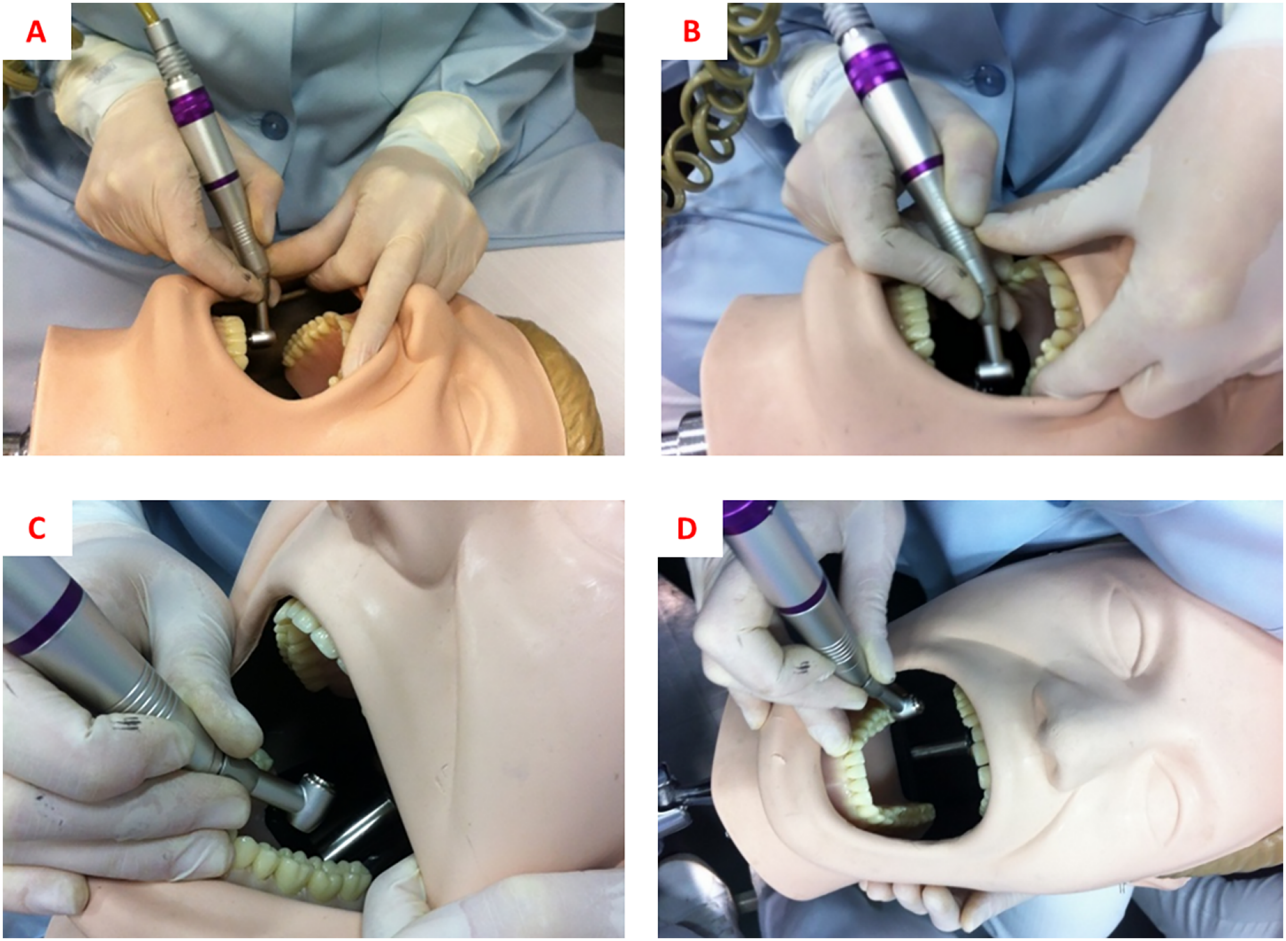
(A) Finger rests for cavity preparation on tooth 16. (B) Finger rests for cavity preparation on tooth 26. (C) Finger rests for cavity preparation on tooth 36. (D) Finger rests for cavity preparation on tooth 46.

For “no finger rest” work, cavity preparations on teeth 16, 26, 36, and 46 were performed with the operator working in a 9 or 11 o’clock position, without supporting the inactive fingers (ring and minimum fingers) of the working hand in any location (tooth, mouth, or opposite hand). For the “usual rest position” work, the operator chose the fingers and working positions that were more comfortable and safer, as shown in Table 1 and Fig. 1. For “ergonomic rest position” work, the recommendations of the Ergonomics in Dentistry discipline of the São Paulo State University (UNESP), School of Dentistry, Araraquara (Porto, 1994) were followed, which are presented in Table 1 and Fig. 2.

**Table 1:** Description of working position and fingers for “ergonomic rest position” and “usual rest position”.

	Ergonomic rest position			Usual rest position		
Tooth	Working position	Working hand (R)	Support hand (L)	Working position	Working hand (R)	Support hand (L)
16	9 h	-In abduction -Rested on the thumb of the left hand by the micromotor	-In abduction -Middle finger extended resting on the vestibular of the left upper central and lateral incisors	9 h	-In abduction -Rested on the thumb of the left hand by the micromotor	-In abduction -Index finger flexed moving cheek away on the right side
26	9 h	-In abduction -Rested on the thumb of the left hand by the micromotor	-In adduction -Index finger extended resting on the vestibular of the left upper premolars	9 h	-In abduction -Rested on the index finger of the left hand by the micromotor	-Neutral position -Thumb flexed moving the cheek away on the left side and index finger extended supporting micromotor head
36	11 h	-In adduction -Ring finger semi flexed resting on lower left lateral incisor and canine	-In adduction -Index finger flexed moving cheek away on the left side	11 h	-In abduction -Ring finger extended resting on chin	-In adduction -Thumb extended supporting micromotor head
46	11 h	-In adduction -Ring finger flexed resting on lower right central and lateral incisors	Free	11 h	-In abduction -Ring finger extended resting on right lower first molar	-In adduction -Thumb extended supporting micromotor head

### Muscular activity

For the analysis of muscle activity, surface EMG was used, which analyzed muscles of the neck (right sternocleidomastoid), shoulder (right anterior deltoid), thorax (right pectoralis major), arm (right biceps), forearm (right brachioradialis), and hand (right abductor pollicis).

This study followed the protocol recommendations for non-invasive surface EMG muscle assessment–SENIAM (Hermens et al., 2000), as well as the methodology proposed by Pazos et al. (2022). Myosystem-Br1 electromyography (Myosystem Br1; DataHominis Tec. Ltda., Uberlândia, Brazil) was used to record, process, and analyze the EMG conditions, with simultaneous acquisition and surface-active differential electrodes (two 10 mm-long × 2 mm-wide silver chloride bars 10 mm apart) with an input impedance of 1,010 Ω/6 pf, bias current input of ±2 nA, common-mode rejection ratio of 110 dB at 60 Hz, and gain equal to 20×. The EMG signals were further amplified by 50× (total gain 1,000×), band-pass filtered (20 Hz–1 kHz), and sampled at a frequency of 2 kHz with 16 bits resolution. The electrode placement site was prepared by alcohol asepsis at the level of the muscles studied, as well as in the right wrist region, where a ground electrode was placed to work as a reference electrode, ensuring the quality of the signal.

The EMG signals were collected continuously for 120-s period, while the simulated cavity preparation was performed, stored on a computer, and then processed and analyzed (Milerad et al., 1991). This period was established based on the average time required to perform class I cavity preparations. Electromyographic data were collected during the completion of each preparation. To standardize the electromyographic signal, the operators performed maximum voluntary contractions (MVC) against manual resistance for 4 s with each muscle group under study (Haddad et al., 2012). The operator sat on a dental stool in a comfortable position, and the MVC was performed against manual resistance by a laboratory technician who had experience in EMG data collection. The operator took a 10-min break before beginning the cavity preparations (Pejcić et al., 2016). Data normalization was performed to allow comparisons between different levels of muscle activity. A calculation was made for this by dividing the EMG RMS (Root Mean Square) value by the EMG RMS (root mean square) value during MVC.

### Angular deviation

The angular deviation from the neutral position was based on the RULA method (McAtamney & Nigel Corlett, 1993), as well as the recommendations of the Ergonomics in Dentistry, São Paulo State University (UNESP), School of Dentistry, Araraquara (Porto, 1994). To analyze this, the working postures were recorded during the entire cavity preparation procedure using three digital cameras (GoPro Hero 4) positioned on the tripods. The tripods were placed at 1 m away from the operator and 70 cm apart, thereby providing adequate visualization of all parts of the body to be evaluated.

The measurement of angular deviations of the neck, trunk, arm, and forearm were performed by a properly calibrated and blinded researcher using “Software for Postural Assessment” (SAPO), version 0.69. In this software, a reference point is placed for the two lines traced. Vertical line represents the neutral position of the region to be evaluated, whereas the oblique line to the reference point follows the actual inclination adopted by the operator. The software then calculates the numerical value that represents the angle formed by these three points (Wajngarten & Garcia, 2019).

The researcher’s calibration was conducted during a pilot study. It consisted of evaluating 10 angular deviations of each region in duplicate, with an interval of 1 week between assessments. Intra-examiner agreement was calculated using the Intraclass Correlation Coefficient (ρ), where *ρ* = 0.864 and *p* < 0.001 were obtained.

### Statistical analysis

Descriptive statistical analyses were performed using IBM SPSS Statistics version 27. Normality was verified using skewness ≤3 and kurtosis ≤7 (Marôco, 2010). The homoscedasticity assumption was verified using Levene’s test (*p* = 0.001–0.928 for muscular activity and *p* = 0.010–0.907 for angular deviation). A one-factor analysis of variance (ANOVA) was then performed, in which only the finger rest position was considered a factor. A tooth cannot be included as a second factor in the same analysis, since the positions for each tooth are different, and consequently, the muscle activity and angular deviation are not comparable (see Table 1 and Figs. 1 and 2). Multiple comparisons were then performed using Tukey’s or Games-Howell’s *post-hoc* tests (depending on the condition of homoscedasticity and heteroscedasticity). The significance level adopted was 5%.

## Results

Table 2 presents the mean, standard deviation, and summary of the one-factor ANOVA of the normalized EMG values of the sternocleidomastoid, deltoid, pectoralis major, biceps brachii, brachioradialis, and right abductor pollicis muscles during cavity preparations in the first molars, according to the type of finger rest position.

**Table 2:** Mean, standard deviation and summary of the one-factor ANOVA of the EMG normalized values (mV) of the sternocleidomastoid, deltoid, pectoralis major, biceps brachii, brachioradialis and abductor pollicis right muscles during the performance of cavity prep.

Tooth	Finger rest positions									
	No finger rest	Usual rest position	Ergonomic rest position	SS	df	MQ	F	π	η2	*p*
**Sternocleidomastoid**										
16	53.596 ± 20.496	44.965 ± 14.246	45.817 ± 17.216	452.504	2	226.252	0.738	0.162	0.052	0.487
26	46.193 ± 25.095	49.874 ± 18.184	40.752 ± 19.602	421.234	2	210.617	0.470	0.119	0.034	0.630
36	47.483 ± 24.435	45.724 ± 19.832	47.482 ± 28.242	20.620	2	10.310	0.017	0.052	0.001	0.983
46	47.081 ± 26.570	50.831 ± 23.851	46.222 ± 26.435	120.159	2	60.079	0.091	0.063	0.007	0.913
**Deltoid**										
16	8.724 ± 1.762	7.673 ± 2.636	9.036 ± 2.438	10.212	2	5.106	0.957	0.063	0.007	0.397
26	9.335 ± 2.255	8.713 ± 2.209	7.312 ±3.034	21.472	2	10.736	1.679	0.322	0.111	0.205
36	12.812 ± 3.019**A**	6.240 ± 3.928**B**	5.850 ± 3.155**B**	306.090	2	153.045	13.306	0.995	0.496	<0.001
46	8.806 ± 2.004	7.373 ± 3.113	7.645 ± 3.140	11.594	2	5.797	0.738	0.162	0.052	0.488
**Pectoralis major**										
16	66.409 ± 17.055**A**	48.615 ± 33.543**AB**	38.705 ± 23.994**B**	3,941.164	2	1,970.582	2.968	0.529	0.180	0.027
26	57.786 ± 21.803**A**	43.074 ± 14.169**AB**	37.513 ± 11.786**B**	2,194.455	2	1,097.227	4.039	0.670	0.230	0.029
36	50.843 ± 17.566	44.994 ± 14.451	39.462 ± 11.439	647.814	2	323.907	1.499	0.291	0.100	0.241
46	42.489 ± 13.161	39.952 ± 15.114	34.306 ± 11.449	374.684	2	187.342	1.055	0.215	0.072	0.362
**Biceps brachii**										
16	0.134 ± 0.027**A**	0.663 ± 0.156**B**	0.096 ± 0.057**AB**	0.023	2	0.012	7.975	0.932	0.371	0.002
26	0.151 ± 0.027**A**	0.072 ± 0.024**B**	0.095 ± 0.053**B**	0.033	2	0.016	11.563	1.000	0.461	<0.001
36	0.325 ± 0.073**A**	0.246 ± 0.122**AB**	0.216 ± 0.079**B**	0.063	2	0.031	3.511	0.605	0.206	0.044
46	0.254 ± 0.064**A**	0.102 ± 0.036**B**	0.130 ± 0.097**B**	0.130	2	0.065	12.974	0.994	0.490	<0.001
**Brachioradialis**										
16	0.168 ± 0.037**A**	0.108 ± 0.306**B**	0.106 ± 0.235**B**	0.025	2	0.013	13.093	0.994	0.492	<0.001
26	0.157 ± 0.026**A**	0.117 ± 0.021**B**	0.106 ± 0.206**B**	0.015	2	0.007	13.575	0.996	0.501	<0.001
36	0.171 ± 0.031	0.137 ± 0.026	0.157 ± 0.513	0.006	2	0.003	2.089	0.391	0.134	0.143
46	0.162 ± 0.023	0.142 ± 0.270	0.142 ± 0.290	0.003	2	0.001	1.610	0.310	0.107	0.219
**Abductor pollicis**										
16	1.236 ± 0.376**A**	0.938 ± 0.378**AB**	0.813 ± 0.259**B**	0.946	2	0.473	4.036	0.670	0.230	0.029
26	2.400 ± 0.363**A**	1.562 ± 1.016**B**	0.834 ± 0.364**B**	12.279	2	6.139	14.206	0.997	0.513	<0.001
36	1.814 ± 0.482**A**	1.086 ± 0.535**B**	1.209 ± 0.609**AB**	3.034	2	1.517	5.110	0.777	0.275	0.013
46	1.633 ± 0.516	1.207 ± 0.508	1.118 ± 0.479	1.519	2	0.760	3.021	0.537	0.183	0.065

**Notes:**

Tukey’s or Games-Howell’s *post-hoc* tests.

A, B, AB, Equal capital letters in bold indicate statistical similarity between the compared groups (columns), while different capital letters indicate statistical difference between the groups (columns).

As shown in Table 2, there was no statistically significant difference in the muscular activity of the right sternocleidomastoid muscle, according to the different types of finger rest positions during cavity preparation of teeth 16 (*p* = 0.487), 26 (*p* = 0.630), 36 (*p* = 0.983), and 46 (*p* = 0.913).

For the right anterior deltoid muscle, work with no finger rest resulted in greater and significantly different muscle activity when compared with work in ergonomic and usual rest positions (Table 2).

Regarding the right pectoralis major and brachioradialis muscles, it was observed that work in an ergonomic rest position showed lower and significantly different muscle activity from work with no finger rest for both teeth 16 (*p* = 0.027 and *p* < 0.001, respectively) and 26 (*p* = 0.029 and *p* < 0.001, respectively) (Table 2).

For the right biceps brachii muscle, higher activity was observed for work with no finger rest in both arches, thereby having a significant difference between the usual rest position in tooth 16 (*p* < 0.001), usual and ergonomic rest positions in teeth 26 (*p* < 0.001) and 46 (*p* < 0.001), and ergonomic rest position in tooth 36 (*p* = 0.044) (Table 2).

Regarding the right abductor pollicis muscle, higher muscle activity was observed during work with no finger rest on teeth 16 (*p* = 0.029), 26 (*p* < 0.001), and 36 (*p* = 0.013) (Table 2).

Table 3 presents the mean, standard deviation, and summary of the one-factor ANOVA of the mean angular deviation from the neutral position of the neck, trunk, arm, and forearm observed during the performance of cavity preparations in the first molars, according to the type of finger rest position.

**Table 3:** Mean, standard deviation and summary of the One-factor ANOVA of the mean angular deviation (°) from the neutral position of the neck, trunk, arm and forearm observed during the performance of cavity preparations in first molars, according to the type of f.

Tooth		Finger rest positions									
		No finger rest	Usual rest position	Ergonomic rest position	SS	df	MQ	F	π	η2	*p*
**Neck**											
16		38.93 ± 5.59	34.54 ± 4.18	35.03 ± 1.93	115.741	2	57.870	3.304	0.577	0.197	0.052
26		39.21 ± 6.88	33.97 ± 5.19	36.33 ± 6.07	137.739	2	68.869	1.859	0.353	0.121	0.175
36		36.58 ± 6.66**A**	32.03 ± 5.98**AB**	29.69 ± 4.86**B**	245.501	2	122.750	3.550	0.610	0.208	0.043
46		33.37 ± 3.28	30.84 ± 7.71	30.89 ± 4.60	41.846	2	20.923	0.687	0.154	0.048	0.512
**Trunk**												
16		6.04 ± 2.25	4.27 ± 1.74	5.34 ± 1.36	15.893	2	7.946	2.389	0.440	0.150	0.111
26		7.12 ± 1.65	6.01 ± 0.99	5.78 ± 1.53	10.269	2	5.134	2.545	0.465	0.159	0.097
36		9.48 ± 2.62	9.53 ± 2.01	8.85 ± 1.62	2.873	2	1.436	0.318	0.095	0.023	0.730
46		7.94 ± 2.28	8.51 ± 1.89	7.92 ± 1.40	2.245	2	1.122	0.314	0.095	0.023	0.733
**Arm**											
16		15.17 ± 4.84**A**	8.33 ± 3.51**B**	7.54 ± 2.52**B**	352.089	2	176.044	12.562	0.992	0.482	<0.001
26		12.03 ± 2.80**A**	9.48 ± 3.05**AB**	7.89 ± 2.50**B**	87.234	2	43.617	5.590	0.815	0.293	0.009
36		5.82 ± 2.66	4.05 ± 2.37	3.78 ± 1.96	24.558	2	12.279	2.227	0.414	0.142	0.127
46		8.20 ± 3.10**A**	7.07 ± 2.24**AB**	5.27 ± 1.86**B**	43.673	2	21.836	3.622	0.619	0.212	0.040
**Forearm**											
16		12.37 ± 3.69	17.22 ± 8.31	16.59 ± 5.11	139.093	2	69.546	1.916	0.362	0.124	0.167
26		11.50 ± 5.74	10.11 ± 5.31	13.60 ± 5.07	61.741	2	30.870	1.065	0.217	0.073	0.359
36		4.38 ± 2.15	3.82 ± 1.79	6.05 ± 2.09	26.918	2	13.459	3.302	0.577	0.197	0.052
46		5.72 ± 3.80	6.36 ± 5.64	8.36 ± 3.73	37.931	2	18.965	0.946	0.196	0.065	0.401

**Notes:**

Tukey’s or Games-Howell’s *post-hoc* tests.

A, B, AB, Equal capital letters in bold indicate statistical similarity between the compared groups (columns), while different capital letters indicate statistical difference between the groups (columns).

A significant difference was observed (Table 3) only for the angular deviation from the neutral position of the arm for teeth 16 (*p* < 0.001), 26 (*p* = 0.009), and 46 (*p* = 0.040). In tooth 16, a larger angular deviation from the neutral position of the right arm was observed when performing cavity preparations with no finger rest, thereby differing significantly from the cavity preparation performed with the usual and ergonomic rest positions. For teeth 26 and 46, the ergonomic rest position provided a lower angular deviation from the neutral position of the right arm, which was significantly different from the cavity preparation performed with no finger rests.

Only angular deviation from the neutral neck position was significant for tooth 36 (*p* = 0.043). It was observed that the angular deviation of the neutral position of this region was lower during the performance of the cavity preparations with the ergonomic rest position, thereby differing significantly from the cavity preparation performed with no finger rest (Table 3).

## Discussion

Performing dental work with finger rest is an alternative that can help prevent musculoskeletal disorders (Dong et al., 2005; Cosaboom-FitzSimons et al., 2008). Thus, the present study aimed to observe the effect of different finger rest positions on muscle activity and angular deviation from the neutral position of the upper extremity of the body during pre-clinical cavity preparations.

In this study, the types of finger rest evaluated were “no finger rest”, “usual finger rest position” used by the operator during clinical routine, and “ergonomic finger rest position”, which was proposed by Porto (1994) and has been taught for many years at the São Paulo State University (Unesp), School of Dentistry, Araraquara. In the Porto (1994)’s proposal, the oral cavity is divided into six parts, which are called sextants. For each sextant, the operator must adopt an appropriate working position (9 or 11 o’clock), position the patient’s head according to the worked arch (up or down, to the left or to the right), and use the fingers and/or free hand as a rest to provide stability and firmness in the working hand, and consequently, less contraction of the muscles involved in dental performance, such as the muscles from hand, forearm, arm, shoulder and thorax.

It was not found in the literature whether this strategy of working with finger rest position is taught in other dental schools. Few works were found on the philosophy of working with finger rest position, including Dong et al. (2005) and Cosaboom-FitzSimons et al. (2008). Therefore, our group decided to test the Porto’s philosophy.

It was possible to observe that the muscular activity of the neck (right sternocleidomastoid) was not influenced by the support of the fingers. In contrast, work with ergonomic rest position on tooth 36 promoted less muscle activity in the shoulder region (right deltoid) when compared to work without finger rest. Because this tooth is located on the left side of the lower arch, it is further away from the right-handed operator; therefore, work with no finger rest may have required greater support from the right arm. This support process is guaranteed by shoulder abduction (Kronberg, Németh & Broström, 1990), a movement that requires contraction of the deltoid muscle and generates higher muscle activity in this region.

In the muscles of the upper thorax (right pectoralis major), the performance on teeth 16 and 26 also showed lower activity in the ergonomic rest position. Porto (1994) recommended that in the upper posterior region, the motor should rest on the thumb of the left hand and the middle finger of the hand on the vestibular surface of the upper anterior teeth. This may have helped in load distribution, and consequently allowed for lower muscle activity. In contrast, for cavity preparation of the first molars of the upper arch, the elbow may approach the trunk because of arm adduction movement (Neumann, 2011). This requires the recruitment of muscle groups, such as the pectoralis major, especially when working with no finger rest.

Work with an ergonomic rest position is taught and trained by students in the pre-clinical training phase on dental phantom heads. However, when they start clinical care, dental treatment is performed on real patients where an error could cause harm their oral or general health (Prince et al., 2005; Hell et al., 2008; Haralur & Al-Malki, 2014). Therefore, students initially have difficulties in applying ergonomic rest positions, and they end up developing the usual rest position. Thus, because working in a finger rest position is part of the student’s routine, performing preparations without support may cause greater contraction in the shoulder and thorax region (Villanueva, Dong & Rempel, 2007).

We observed that working with no finger rest resulted in higher activity of the arm muscle (right biceps brachii), regardless of the tooth prepared. In this situation, the forearm may not have stayed close to the body during work without support, and this less-pronated position required greater contraction of the biceps to promote hand stability, and consequently, higher muscle activity (Dong et al., 2007; Neumann, 2011; Villanueva, Dong & Rempel, 2007).

Higher muscular activity of the right brachioradialis muscle was observed in work with no finger rest only in the teeth of the superior arch. Because this muscle is the main flexor of the elbow and its maximum activity occurs when there is a movement of total flexion of the joint (Basmajian & Latif, 1957; Neumann, 2011), the greater flexion of the elbow to work in this region may have generated an increase in activity of this muscle.

The abductor pollicis muscle, which is an intrinsic muscle of the hand, allows the thumb to be placed in different positions to help hold instruments and fine control the fingers by combining the elements of abduction, flexion, and medial rotation in the joint (Kaufman et al., 1999). In this study, the abductor pollicis muscle showed higher activity when working with no finger rest on teeth 16, 26, and 36. This fact can be explained by the necessity of this musculature to work both in the movement precision and in the hand stability during the execution of the procedure, since it was not supported on any other surface (Dong et al., 2005). However, the activity of the abductor pollicis muscle was not influenced by the different rest positions during cavity preparation on tooth 46. This may be related to the fact that this tooth is closest to the right-handed operator, thereby providing greater hand stability.

In the analysis of angular deviations from neutral positions, cavity preparation of teeth 16, 26, and 46 with no finger rest resulted in a higher angular deviation only in the arm. Meanwhile, work with no finger rest on tooth 36 resulted in greater angular deviation in the neck. As this tooth was in the lower arch and on the left side, it was further away from the right-handed operator, which may have required a greater head tilt for better visualization.

The results obtained in this study are important for occupational health in dentistry, as they scientifically confirmed that the use of non-active fingers for stabilization during the execution of dental work resulted in less muscle stress in the upper limbs. Thus, this strategy may be adopted to prevent musculoskeletal disorders in dentistry. According to Dong et al. (2005), the use of a finger rest can improve work precision and prevent sudden movements.

In general, the results of this study showed that if the operator does not necessarily use the ergonomic rest position, but adopts a form of rest for the fingers that is more comfortable and compatible with their needs, they will be working with less muscle activity in the region of the upper limbs, as well as less angular deviation from the neutral position. Although many students give up the ergonomic rest position when starting clinical care, its teaching and training is necessary because it allows them to develop a personalized rest position, which becomes habitual. These data provide information to professors in the field of ergonomics in dentistry to adopt the teaching of finger rest position for students during their pre-clinical phase, which will develop in their students the awareness of its importance for the maintenance of occupational health.

A limitation of this study is the laboratory design and performance of the simulated and standardized procedures. This care was taken to control possible unforeseen events that could occur during the care of a real patient. Thus, future studies can be conducted in a real clinical environment based on the results obtained in a controlled environment. Despite this, considering the scarcity of studies on this topic, this study makes an important contribution to the field of education in dentistry.

We believe that a strength of our study is the performance of electromyographic measurements and filming during the complete procedure instead of small collection cycles. We chose to conduct the methodology in this way because we believe that this would bring more reliable results and information to clinical reality and the environment.

## Conclusions

In general, the use of non-active finger rests during simulated cavity preparations, regardless of the type of rest position, provided less muscle activity and angular deviation from the neutral position of the upper extremity of the body during pre-clinical activities.

## Supplemental Information

10.7717/peerj.15663/supp-1 Supplemental Information 1 Raw Data. All electromyography and angular deviation measurements.

## Additional Information and Declarations

### Data Availability

The following information was supplied regarding data availability: The raw data are available in the Supplemental Files.

## References

[ref-1] Alhazzazi TY, Alzebiani NA, Alotaibi SK, Bogari DF, Bakalka GT, Hazzazi LW, Jan AM, McDonald NJ (2016). Awareness and attitude toward using dental magnification among dental students and residents at King Abdulaziz University, Faculty of Dentistry. BMC Oral Health.

[ref-2] Arnett MC, Gwozdek AE, Ahmed S, Beaubien HD, Yaw KB, Eagle IT (2017). Assessing the use of loupes and lights in dental hygiene educational programs. Journal of Dental Hygiene.

[ref-3] Basmajian JV, Latif A (1957). Integrated actions and functions of the chief flexors of the elbow: a detailed electromyographic analysis. The Journal of Bone & Joint Surgery.

[ref-4] Cervera-Espert J, Pascual-Moscardó A, Camps-Alemany I (2018). Wrong postural hygiene and ergonomics in dental students of the University of Valencia (Spain) (part I). European Journal of Dental Education.

[ref-5] Corrocher PA, Presoto CD, Campos JA, Garcia PP (2014). The association between restorative pre-clinical activities and musculoskeletal disorders. European Journal of Dental Education.

[ref-6] Cosaboom-FitzSimons ME, Tolle SL, Darby ML, Walker ML (2008). Effects of 5 different finger rest positions on arm muscle activity during scaling by dental hygiene students. Journal of Dental Hygiene.

[ref-7] Dable RA, Wasnik PB, Yeshwante BJ, Musani SI, Patil AK, Nagmode SN (2014). Postural assessment of students evaluating the need of ergonomic seat and magnification in dentistry. The Journal of Indian Prosthodontic Society.

[ref-8] Diaz-Caballero AJ, Gómez-Palencia IP, Díaz-Cárdenas S (2010). Ergonomic factors that cause the presence of pain muscle in students of dentistry. Medicina Oral Patología Oral y Cirugia Bucal.

[ref-9] Dong H, Barr A, Loomer P, Rempel D (2005). The effects of finger rest positions on hand muscle load and pinch force in simulated dental hygiene work. Journal of Dental Education.

[ref-10] Dong H, Loomer P, Villanueva A, Rempel D (2007). Pinch forces and instrument tip forces during periodontal scaling. Journal of Periodontology.

[ref-11] Garcia PPNS, Pinelli C, Derceli JR, Campos JADB (2012). Musculoskeletal disorders in upper limbs in dental students: exposure level to risk factors. Brazilian Journal of Oral Sciences.

[ref-12] Garcia PP, Pugliesi PM, Wajngarten D, Td Neves, Pazos JM, Dovigo LN (2021). Development and assessment of an indirect vision training programme for operatory dentistry: effects on working posture. European Journal of Dental Education.

[ref-13] Garcia PPNS, Wajngarten D, Campos JADB (2018). Development of a method to assess compliance with ergonomic posture in dental students. Journal of Education and Health Promotion.

[ref-14] Graça CC, Araújo TM, Silva CMP (2006). Desordens musculoesqueléticas em cirurgiões-dentistas. Sitientibus.

[ref-15] Haddad O, Sanjari MA, Amirfazli A, Narimani R, Parnianpour M (2012). Trapezius muscle activity in using ordinary and ergonomically designed dentistry chairs. The International Journal of Occupational and Environmental Medicine.

[ref-16] Haralur SB, Al-Malki AE (2014). Student perception about efficacy of preclinical fixed prosthodontic training to facilitate smooth transition to clinical context. Journal of Education and Health Promotion.

[ref-17] Hayes MJ, Rogers AA, Chuanon J, Tan T, Lai I, Yong E (2021). Dental and oral health students’ perceptions of loupes. International Journal of Occupational Safety and Ergonomics.

[ref-18] Hell EAV, Kuks JB, Schönrock-Adema J, van Lohuizen MT, Cohen-Schotanus J (2008). Transition to clinical training: influence of pre-clinical knowledge and skills, and consequences for clinical performance. Medical Education.

[ref-19] Hermens HJ, Freriks B, Disselhorst-Klug C, Rau G (2000). Development of recommendations for SEMG sensors and sensor placement procedures. Journal of Electromyography and Kinesiology.

[ref-36] Hoerler SB, Branson BG, High AM, Mitchell TV (2012). Effects of dental magnification lenses on indirect vision: a pilot study. Journal of Dental Hygiene.

[ref-20] Kaufman KR, An K-N, Litchy WJ, Cooney WP, Chao EYS (1999). In-vivo function of the thumb muscles. Clinical Biomechanics.

[ref-21] Kronberg M, Németh G, Broström LA (1990). Muscle activity and coordination in the normal shoulder. An electromyographic study. Clinical Orthopaedics and Related Research.

[ref-22] Marôco J (2010). Análise estatística com o PASW Statistics (ex-SPSS).

[ref-23] McAtamney L, Nigel Corlett E (1993). RULA: a survey method for the investigation of work-related upper limb disorders. Applied Ergonomics.

[ref-24] Milerad E, Ericson MO, Nisell R, Kilbom A (1991). An electromyographic study of dental work. Ergonomics.

[ref-25] Neumann DA (2011). Cinesiologia do aparelho musculoesquelético: fundamentos para reablitação.

[ref-26] Onety GCS, Leonel DV, Saquy PC, Silva GP, Ferreira B, Varise TG, Sousa LG, Verri ED, Siessere S, Semprini M, Nepomuceno VR, Regalo SCH (2014). Analysis of endodontist posture utilizing cinemetry, surface electromyography and ergonomic checklists. Brazilian Dental Journal.

[ref-27] Pazos JM, Regalo SCH, de Vasconcelos P, Campos JADB, Garcia PPNS (2022). Effect of magnification factor by Galilean loupes on working posture of dental students in simulated clinical procedures: associations between direct and observational measurements. PeerJ.

[ref-28] Pejcić N, Jovicić MĐ, Miljković N, Popović DB, Petrović V (2016). Posture in dentists: sitting vs. standing positions during dentistry work--an EMG study. Srpski Arhiv Za Celokupno Lekarstvo.

[ref-29] Plessas A, Bernardes Delgado M (2018). The role of ergonomic saddle seats and magnification loupes in the prevention of musculoskeletal disorders. A systematic review. International Journal of Dental Hygiene.

[ref-30] Porto FA (1994). O consultório odontológico.

[ref-31] Prince KJ, Boshuizen HP, van der Vleuten CP, Scherpbier AJ (2005). Students’ opinions about their preparation for clinical practice. Medical Education.

[ref-32] Rempel D, Lee DL, Dawson K, Loomer P (2012). The effects of periodontal curette handle weight and diameter on arm pain: a four-month randomized controlled trial. The Journal of the American Dental Association.

[ref-33] Samoladas E, Barmpagianni C, Papadopoulos DV, Gelalis ID (2018). Lower back and neck pain among dentistry students: a cross-sectional study in dentistry students in Northern Greece. European Journal of Orthopaedic Surgery & Traumatology.

[ref-34] Villanueva A, Dong H, Rempel D (2007). A biomechanical analysis of applied pinch force during periodontal scaling. Journal of Biomechanics.

[ref-35] Wajngarten D, Garcia PPNS (2019). Effect of magnification devices on dental students’ visual acuity. PLOS ONE.

